# Solitary pulmonary metastasis with cystic airspaces in colon cancer: A rare case report

**DOI:** 10.1515/biol-2025-1149

**Published:** 2025-08-18

**Authors:** Kunliang Guo, Yang Li, Jian Chen

**Affiliations:** Department of Cardiothoracic Surgery, Anqing Municipal Hospital, Anqing, China; Department of Pathology, Anqing Municipal Hospital, Anqing, China

**Keywords:** case report, pulmonary metastasis, cystic airspaces, colorectal cancer

## Abstract

This report presents a case of solitary pulmonary metastasis from colon cancer, characterized by cystic airspaces, which can mimic a second primary lung cancer (LC). Preoperative contrast-enhanced computed tomography in a patient with colon cancer revealed a pulmonary micronodule with a cystic cavity in the right upper lobe. The patient subsequently underwent left-sided hemicolectomy followed by six cycles of chemotherapy. During follow-up, computed tomography demonstrated resolution of the right upper lobe nodule. However, 36 months after chemotherapy, a solid nodule with cystic airspaces appeared in the right upper lobe, exhibiting marginal spiculation, traversing vessels, and enhancement, suggestive of cystic LC. Intraoperative frozen section analysis indicated features of intestinal adenocarcinoma. A right upper lobe wedge resection was performed, and postoperative histopathology confirmed the lesion as metastatic colon adenocarcinoma with cystic airspaces. Solitary pulmonary metastasis from colon cancer is relatively uncommon, particularly with cystic presentation. Clinicians and radiologists should maintain heightened suspicion in such atypical cases to avoid missed or delayed diagnoses.

## 1 Introduction

This work has been reported following the SCARE 2020 guideline [1]. Colorectal cancer (CRC) is the third most prevalent malignancy globally [2] and the second most common cancer in China [3]. It is also a leading cause of cancer-related mortality [4]. The lungs are the second most frequent site of metastasis after the liver [5]. Solitary pulmonary metastasis is infrequent and occurs more commonly in patients with rectal cancer [6]. This report presents dynamic radiological findings from chest computed tomography (CT) of a solitary pulmonary nodule (SPN) in a patient with colon cancer. The images illustrate the lesion’s evolution from an early microcystic nodule to a solid nodule with cystic cavities. Final histopathological analysis confirmed the nodule as metastatic colon adenocarcinoma with cystic airspaces, rather than primary lung malignancy.

**Figure 1:**
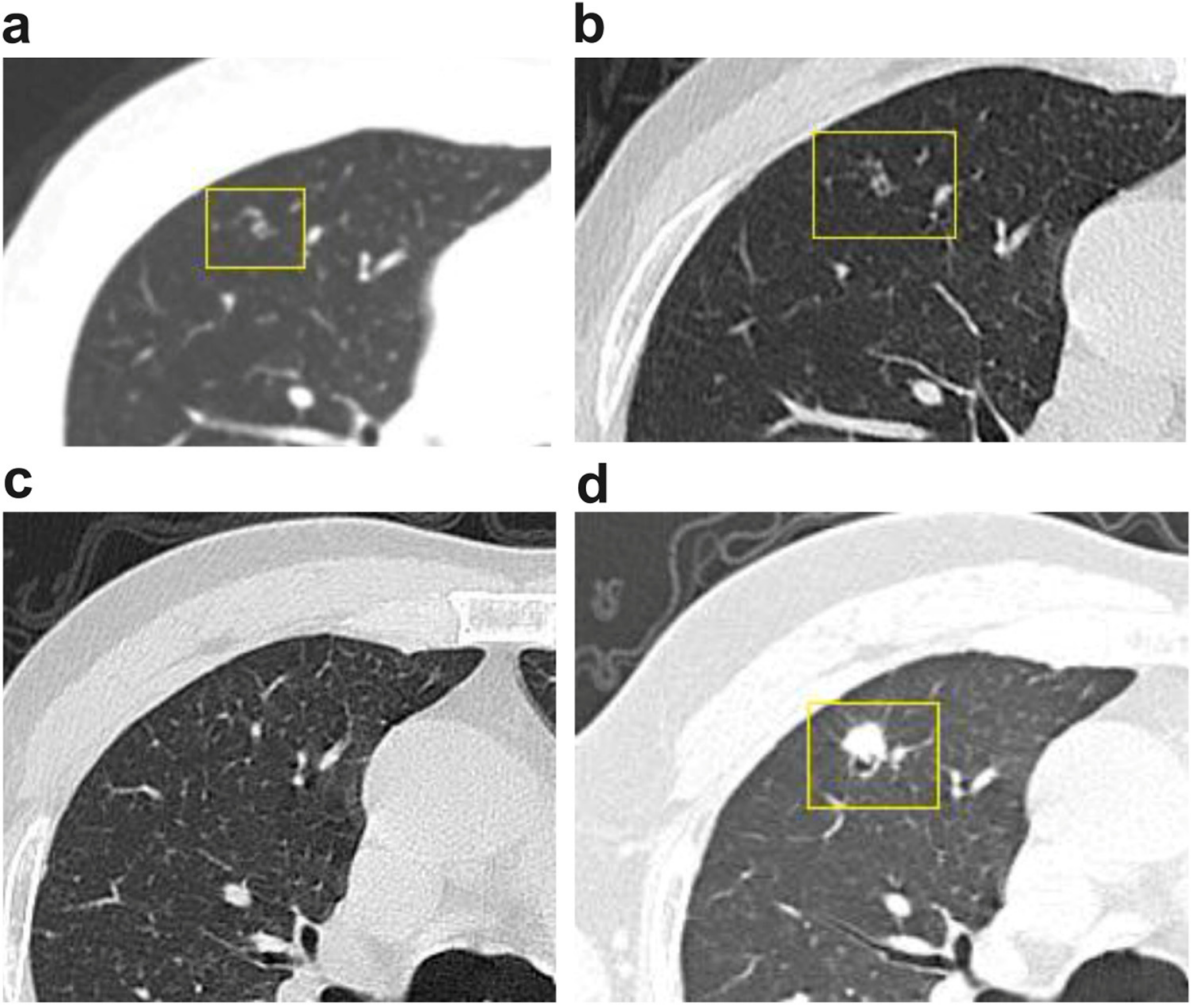
CT scan showing the presence of a cystic airspace in the right upper lobe of the lung on November 18, 2020, (a) and December 17, 2020 (b), the disappearance of the cysts on April 7, 2021 (c), and the presence of a 10-mm-diameter mass with a cystic airspace on April 5, 2024 (d).

In November 2020, a 62-year-old Chinese male presented with abdominal distension and pain without an apparent cause. Following inadequate symptomatic management at a local hospital, he was admitted to our general surgery department 3 months later. The patient had a 2-year history of hypertension, treated with oral amlodipine 5 mg once daily, with good blood pressure control. The patient’s blood pressure was 130/75 mmHg prior to colon cancer surgery. He had a 40-year smoking history averaging 20 cigarettes per day, an appendectomy performed 20 years prior, and an inguinal hernia repair over 10 years ago. The patient denied weight loss, pallor, chills, fever, nausea, or vomiting, reported normal urination, but experienced frequent constipation. He denied any family history of malignancy. The general physical examination, including chest and abdomen, was unremarkable except for an old surgical scar on the abdomen. Preoperative serum carcinoembryonic antigen (CEA), carbohydrate antigen 19-9 (CA19-9), and serum ferritin (SF) levels were within normal limits. Upper abdominal magnetic resonance imaging suggested the possibility of colon cancer. Chest contrast-enhanced computed tomography (CECT) revealed a micronodule in the right upper lobe, measuring 3 mm in diameter, with a central cystic cavity (Figure 1a). The patient underwent laparoscopic resection of the left hemicolon. Histopathology documented moderately differentiated adenocarcinoma without vascular invasion or perineural infiltration. The postoperative diagnosis was stage IIA adenocarcinoma of the left hemicolon. One month after surgery (December 16, 2020), SF and tumor necrosis factor-α levels were elevated, while other tumor markers (including CEA and CA19-9) remained negative. Chest CECT revealed a cystic small nodular opacity in the anterior segment of the right upper lobe, measuring 3 mm in diameter, with well-defined borders and a uniformly thick cyst wall (∼1 mm), without evident mural nodularity (Figure 1b). The patient received two cycles of chemotherapy with oxaliplatin and capecitabine. During the second cycle, he experienced gastrointestinal adverse effects and could not tolerate capecitabine, thus discontinuing treatment. Subsequently, he received four cycles of chemotherapy with oxaliplatin and raltitrexed. Throughout chemotherapy, serological tumor markers remained within normal limits. Before the sixth chemotherapy cycle, chest CECT demonstrated resolution of the lesion in the anterior segment of the right upper lobe (Figure 1c). However, 36 months after chemotherapy completion, chest CECT revealed a solid nodule in the anterior segment of the right upper lobe, approximately 10 mm in diameter, with shallow lobulation, fine spiculation, and marginal cystic formation. Traversing vessels and enhancement were noted within the nodule (Figure 1d). Lung metastatic tumors typically appear on imaging as well-defined solid nodules, with spiculated borders rarely observed. Additionally, all serum tumor markers in the current patient were negative. Taking this into consideration, along with the patient’s medical history, the radiologist diagnosed the tumor as primary cystic lung cancer (LC). The patient and his family declined a preoperative biopsy. Following a multidisciplinary team discussion regarding the malignant potential and resectability of the lung lesion, surgical intervention was recommended. An experienced thoracic surgeon and the surgical team performed a video-assisted thoracoscopic surgery wedge resection of the right upper lung and lymph node biopsy on April 9, 2024. Intraoperative frozen section analysis indicated invasive adenocarcinoma, characterized by columnar cancer cell nuclei without a peripheral lepidic growth pattern. Given the patient’s medical history, metastasis could not be excluded. Consequently, the thoracic surgeons proceeded with the wedge resection.

**Figure 2:**
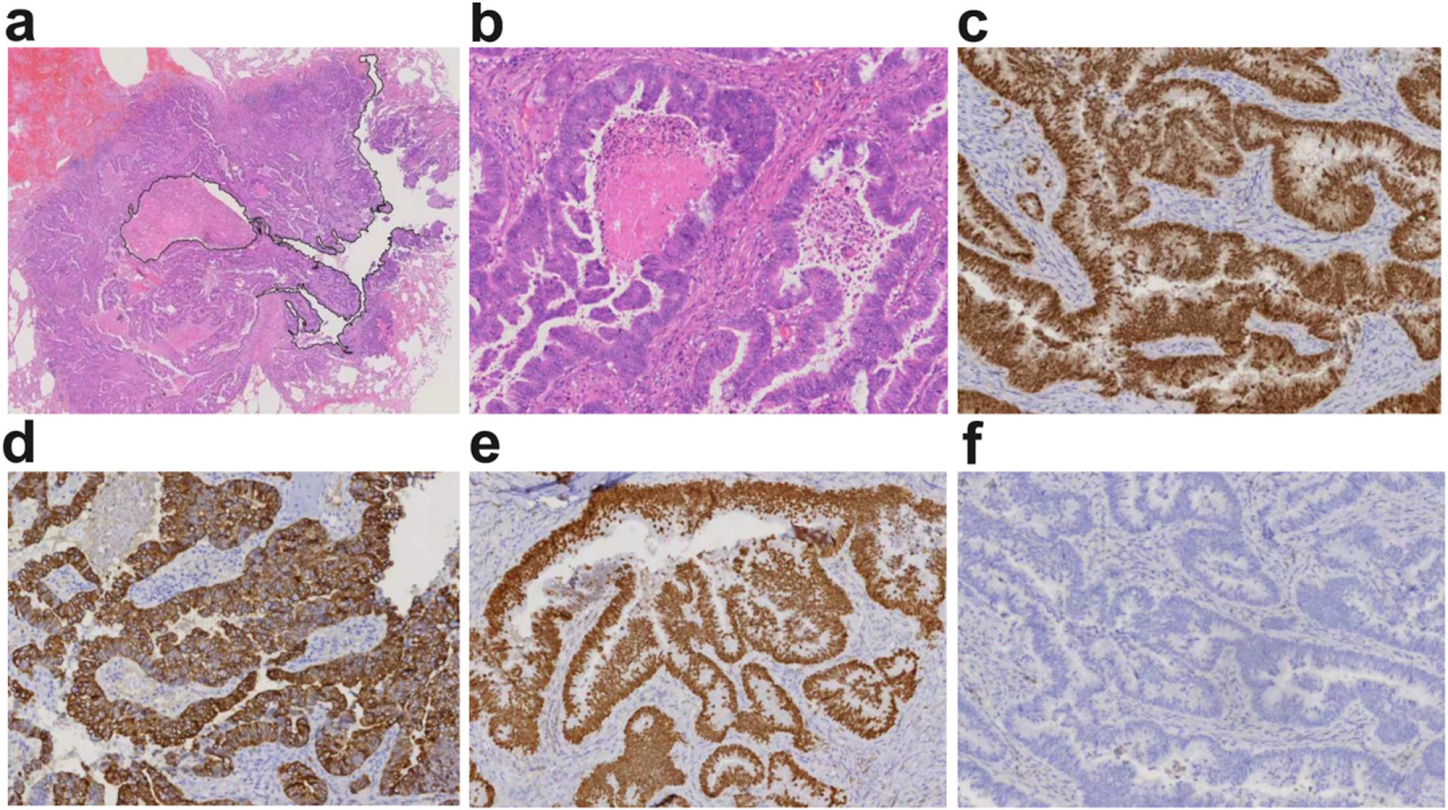
Histopathologic images. The tumor contains a cystic space (a) black line marking; hematoxylin and eosin staining; magnification (×1), with columnar cells, arranged in irregular acinar or sieve-like patterns, with extensive central necrosis (b), hematoxylin and eosin staining; magnification (×10). Immunohistochemically, the tumor was positive for tumor protein CDX-2 (c) EnVision method; magnification (×10), CK-20 (d) EnVision method; magnification (×10), and SATB2 (e) EnVision method; magnification (×10). The tumor was negative for tumor protein TTF-1 (f) EnVision method; magnification (×10).

**Figure 3:**
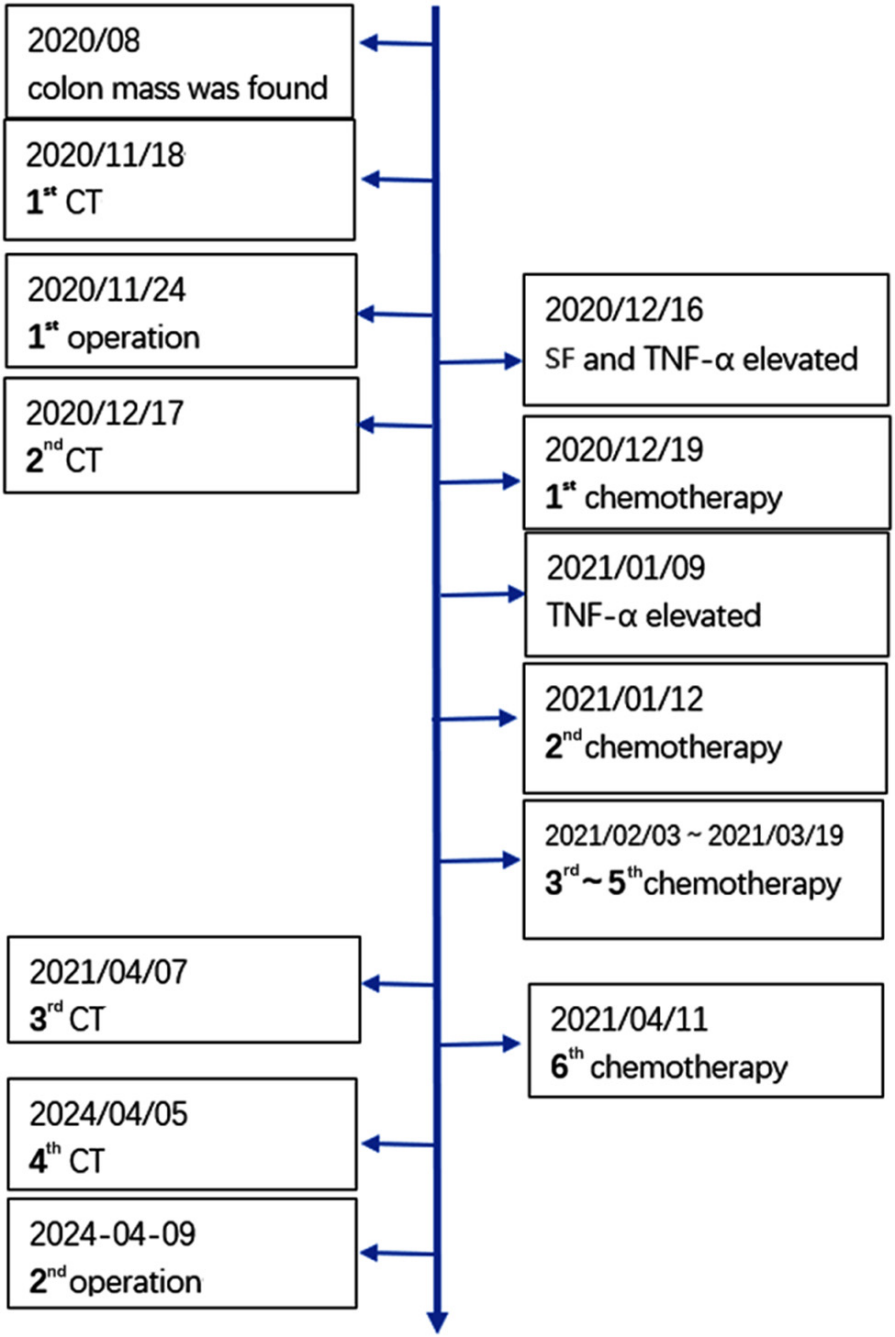
Chronology of patient’s major health events.


Postoperative histopathology revealed irregular large clefts within the cancerous lesion (Figure 2a). The neoplastic cells exhibited columnar morphology with nuclear pseudostratification, arranged in glandular, papillary, or solid patterns. Necrotic debris and nuclear fragmentation were noted within the glandular lumens, consistent with morphological features of colorectal adenocarcinoma. No lepidic growth pattern of adenocarcinoma *in situ* was identified in the surrounding lung parenchyma (Figure 2b). Immunohistochemical staining was positive for CDX-2 (Figure 2c), CK20 (Figure 2d), and SATB-2 (Figure 2e), with membranous expression of β-catenin. TTF-1 (Figure 2f), Napsin-A, and CK7 were negative. Considering the patient’s prior surgical history, these histopathological findings supported metastatic colon adenocarcinoma in the lung, without evidence of vascular invasion, neural involvement, pleural infiltration, or positive surgical margins. No lymph node metastases were detected. The patient recovered uneventfully and was discharged 7 days after surgery. He remained disease-free at the 60-day follow-up (from April 9, 2024). The chronology of the patient major health events is summarized (Figure 3).

**Informed consent:** Informed consent has been obtained from all individuals included in this study.
**Ethical approval:** The research related to human use has been complied with all the relevant national regulations and institutional policies and in accordance with the tenets of the Declaration of Helsinki and has been approved by the Ethical Committee of Anqing Municipal Hospital (2024 No. 147).

## 2 Discussion

Solitary pulmonary metastasis from CRC is uncommon. Data from a large cohort in Singapore showed that the incidence of solitary pulmonary metastasis was 12% in patients with rectal cancer and 6% in those with colon cancer [7]. When an SPN is detected in patients with CRC, it is critical to differentiate between primary LC and solitary pulmonary metastasis (LM) to ensure effective treatment planning and prognostic assessment [8]. Primary LC typically presents radiologically with marginal spiculation, subsolid components, bronchial inflation signs, and pleural changes [9]. In this case, serological tumor markers remained stable. Serial CT scans demonstrated that the SPN, located in the anterior segment of the right upper lobe, initially appeared as a small cystic airspace lesion prior to colon cancer surgery (first and second CT). After five cycles of chemotherapy, the nodule resolved. However, 36 months after completion of the sixth chemotherapy cycle, a solid nodule with cystic airspaces re-emerged in the same location. The radiologist diagnosed the lesion as cystic LC (LC associated with cystic airspaces [LCCA]). Nevertheless, histopathological analysis revealed features characteristic of intestinal adenocarcinoma. Lung-derived markers (TTF-1, Napsin-A, CK7) were negative, whereas gastrointestinal-derived markers (CDX-2, CK20, SATB-2) were positive, and β-catenin showed membranous expression [10,11,12]. The final histopathological diagnosis confirmed metastatic colon adenocarcinoma in the lung.

The term “cystic airspaces” was introduced in 1996 as an expanded peripheral air-containing lung unit surrounded by walls of variable thickness [13]. This definition was refined in 2009 [14]. Pulmonary diseases associated with cystic airspaces include emphysematous bullae, congenital or fibrotic cysts, subpleural blebs, bronchiectatic airways, distal airspace enlargement [15], and the rare entity of primary LCCA [16]. Cavitation in metastatic lung tumors is extremely rare, occurring in only about 4% of cases [17]. Of these, approximately 70% of cases are associated with squamous cell carcinomas originating from the head and neck or cervical regions [17,18]. Adenocarcinomas may arise from various organs, including the pancreas [19], prostate [20], and breast cancers [21]. To date, only four cases of multiple cavitary lung metastases originating from CRC have been reported [22]. In this case, the CT appearance of solitary pulmonary metastasis from colon cancer evolved with disease progression. We hypothesize that two mechanisms may contribute to the formation of early microcystic lesions. One mechanism posits that due to the dual blood supply from pulmonary and bronchial arteries and relatively low blood pressure, a small number of cancer cells shed from the primary tumor disseminate into the pulmonary capillary bed. Some of these cells may evade host defenses, penetrate pulmonary capillaries, and proliferate along the lung septa. Another mechanism suggests that cystic airspaces result from a check-valve phenomenon associated with microscopic malignant lesions [23]. As postoperative chemotherapy progressed, neoplastic cells in the metastatic lesion were significantly reduced by chemotherapeutic agents but not completely eradicated, leading to the temporary disappearance of the lesion on CT. After completing chemotherapy, residual viable cancer cells continued to proliferate, gradually forming invasive growths characterized by intestinal glandular structures of varying sizes. Necrotic material and nuclear debris within these glandular lumens were expelled along the respiratory tract, gradually enlarging the cavity (Figure 2a). This enlarged cavity appeared as a cystic airspace on CT (Figure 1d), leading to misdiagnosis as primary cystic LC by the radiologist.

It is important to note that the occurrence of cavitation in metastatic lung tumors following chemotherapy for CRC is relatively common, which can complicate the diagnostic process. For instance, anti-angiogenic therapies can influence the tumor microenvironment by inhibiting angiogenesis around the tumor, while simultaneously promoting vascularization of the existing tumor vessels. This results in reduced blood flow to the tumor, which lowers the tumor density, thus facilitating the formation of cavitary lesions [24]. However, in this case, when the isolated 3 mm lung nodule was first discovered, the patient had not undergone chemotherapy. Over the course of six cycles of chemotherapy, the lesion disappeared. Thirty-six months after completing chemotherapy, a chest CT scan showed a 10 mm cystic lung nodule in the right lung. The chemotherapy regimen included capecitabine, oxaliplatin, and raltitrexed. The half-life of capecitabine and its metabolites is approximately 45–60 min, oxaliplatin has a half-life of around 240 h, and raltitrexed’s half-life is approximately 198 h. Since the effects of these drugs do not persist for more than 12 months, we conclude that the cavitation of the metastatic lung tumor in this case is unrelated to chemotherapy and is instead a result of the abnormal tumor progression.

Several national and international guidelines (AIOM [25], ESMO [4], and NCCN [26]) recommend surgical resection as the most effective curative treatment for lung metastasis from CRC. The fundamental principle involves completely excising the metastatic lesion while preserving as much healthy lung tissue as possible, to improve patient survival and quality of life. In this case, the intraoperative pathologist identified the distinctive intestinal adenocarcinoma morphology of the lung lesion. Following consultation between thoracic surgeons and pathologists, and considering the patient’s history of colon cancer, they concluded the lesion was more likely metastatic rather than primary pulmonary intestinal adenocarcinoma, prompting a decision to perform wedge resection of the right upper lobe. If the lesion had been misidentified as a second primary cancer and treated by immediate lobectomy, subsequent histopathology confirming metastasis could have led to physician liability and potential medical disputes.

Solitary pulmonary metastasis from colon cancer is rare, particularly when presenting with cystic airspaces, posing a potential diagnostic pitfall. For patients initially diagnosed with CRC, routine chest CT examinations are essential. Furthermore, when cystic lesions are detected on chest CT, especially in patients with prior malignancy, metastatic tumors should be included in the differential diagnosis and management plan, with regular follow-up. Timely diagnosis and surgical intervention remain critical to improving patient prognosis.
